# Supplementary material for: MegaLMM improves genomic predictions in new environments using environmental covariates
Source: Genetics. 2024 Oct 29;229(1):iyae171. doi: 10.1093/genetics/iyae171 (PMC11708919; doi:10.1093/genetics/iyae171)
Supplement: iyae171_Supplementary_Data [file iyae171_supplementary_data.pdf]

SUPPLEMENTAL INFORMATION

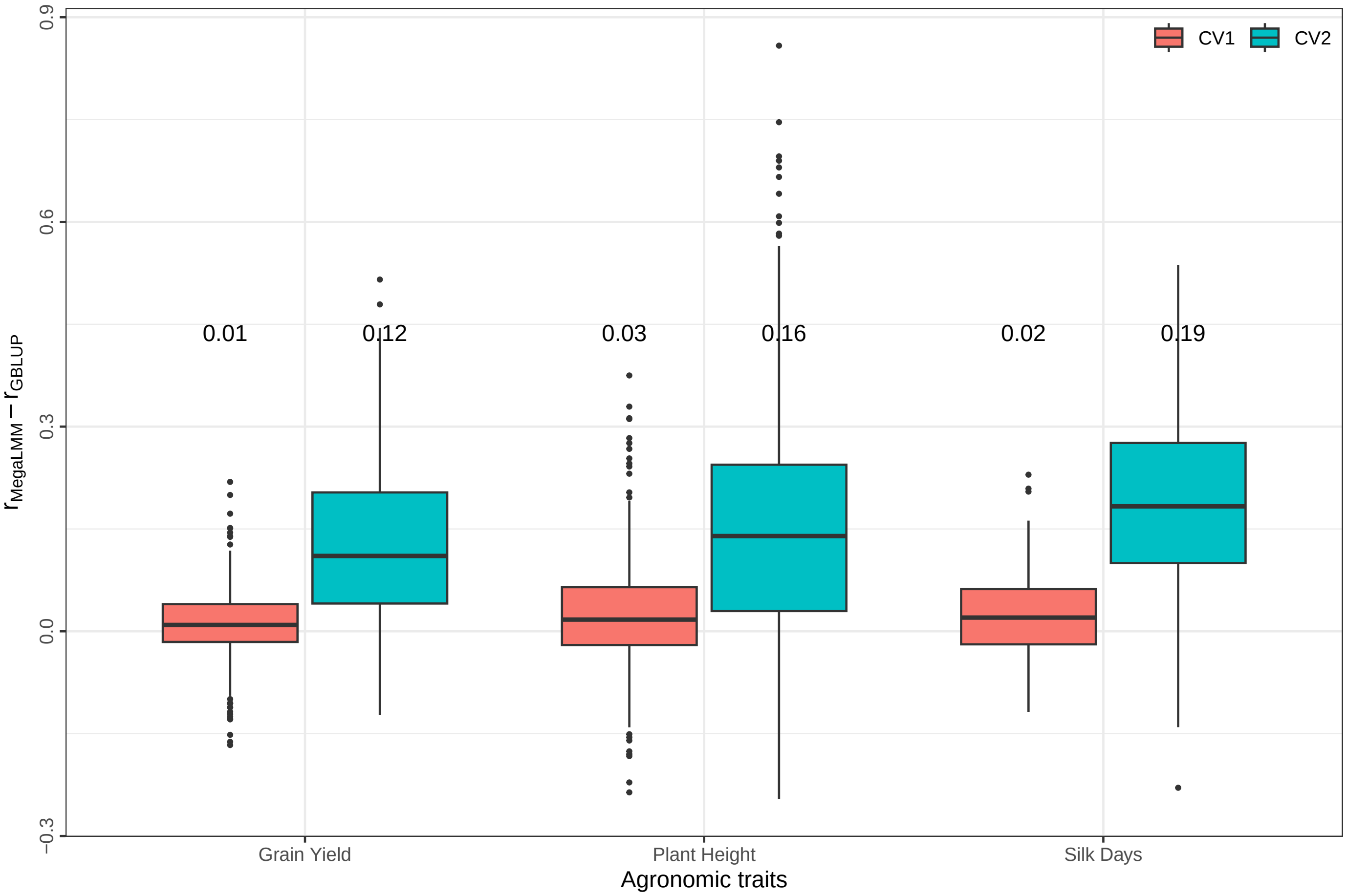

Figure S1. Predictive ability difference between MegaLMM and GBLUP for three agronomic traits (Silk Days, Plant Height, and Grain Yield). Each point within a boxplot represents the predictive ability difference between MegaLMM and GBLUP for a specific experiment. The mean predictive ability difference for each trait within each scenario is shown above the corresponding boxplot.

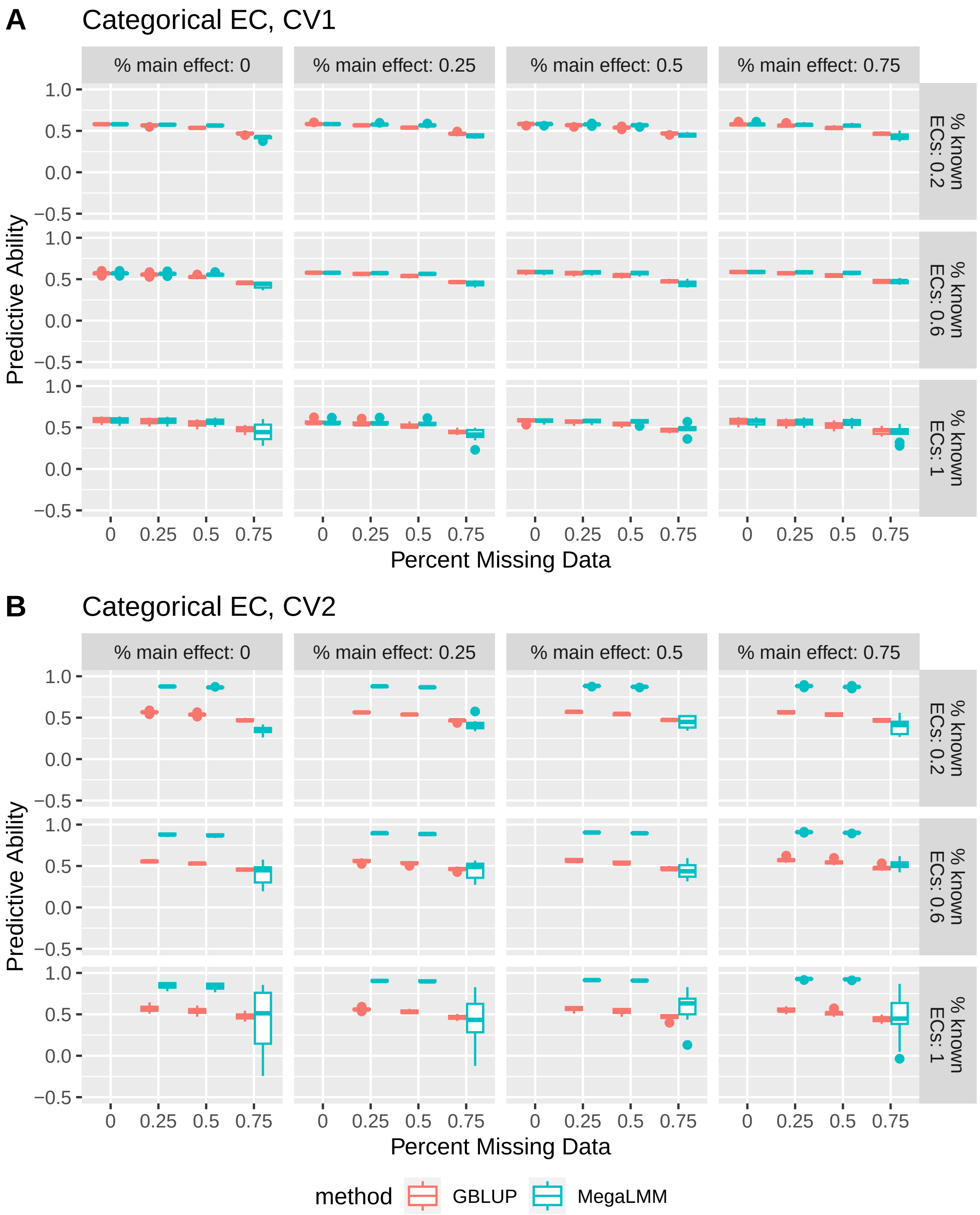

Figure S2. Predictive ability of MegaLMM and GBLUP in training environments in simulations with categorical ECs. Boxplots show predictive abilities (*i.e.* Pearson's correlations between genomic predictions and phenotypes across 10 simulations of each parameter setting. *% main effect* refers to the relative contribution of the positive covariance term across all environments and a covariance term specifying negative covariances among groups of trials. Each scenario included 500 genotypes and 400 trials. A) Average predictive abilities of new genotypes across the 400 trials. B) Average predictive abilities of old genotypes across the 400 trials. Predictive abilities in each trial were calculated only for genotypes that were not observed in that trial but were observed in other trials (*i.e.* sparse testing). Thus *CV2* predictive abilities could not be calculated when the missing data rate was 0%. At 75%, there were four sets of 100 trials that were mutually un-connected, each containing 125 genotypes observed in only those 100 trials and in no others. Results are shown for MegaLMM (blue) and GBLUP (red).

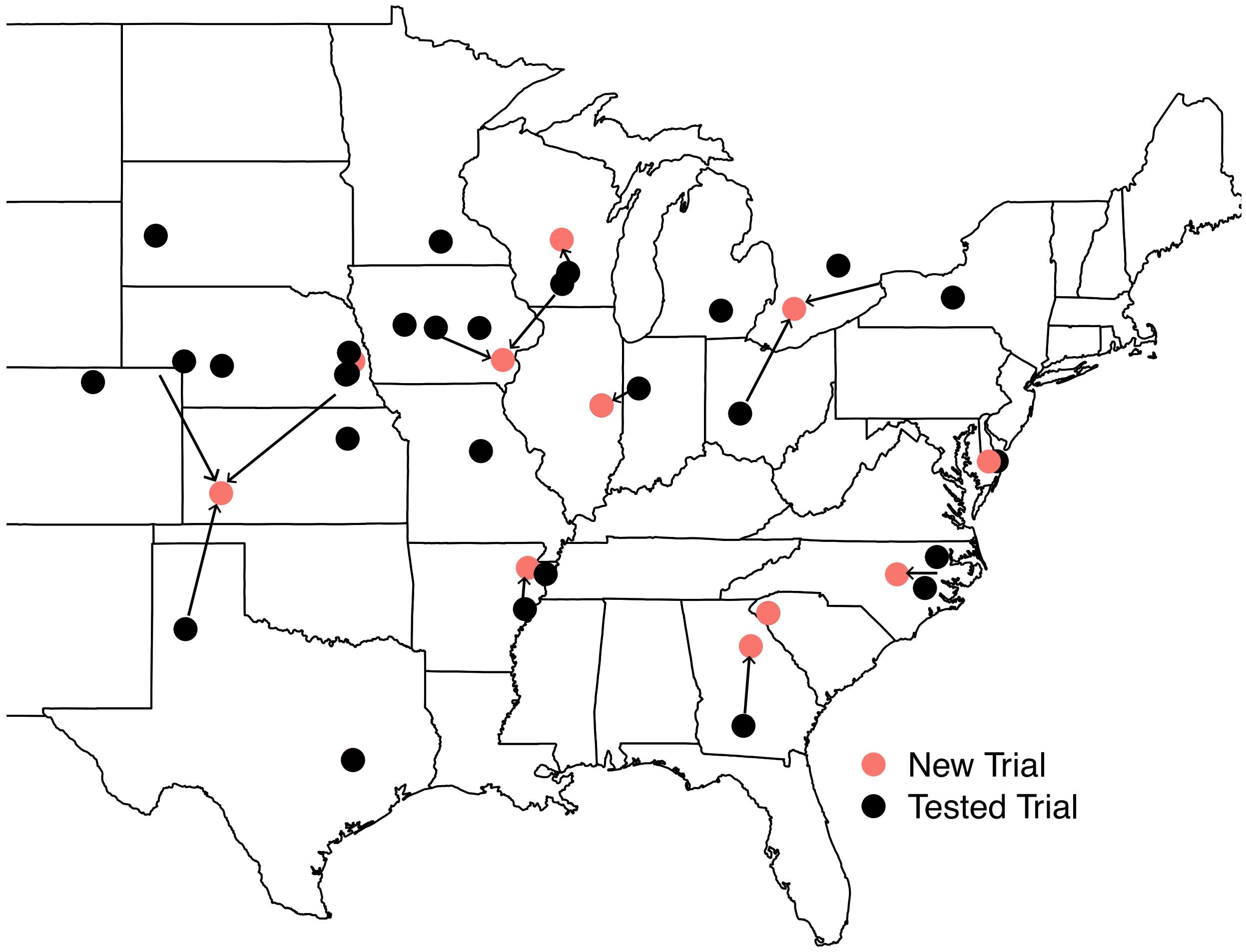

Figure S3. Graphical demonstration of the cross-validation scenario for NewTrial. The red circles represent new trials where phenotypic data was concealed, and the black circles represent tested trials. The tested trials were used to predict the trait performance of experimental genotypes in the new trials.

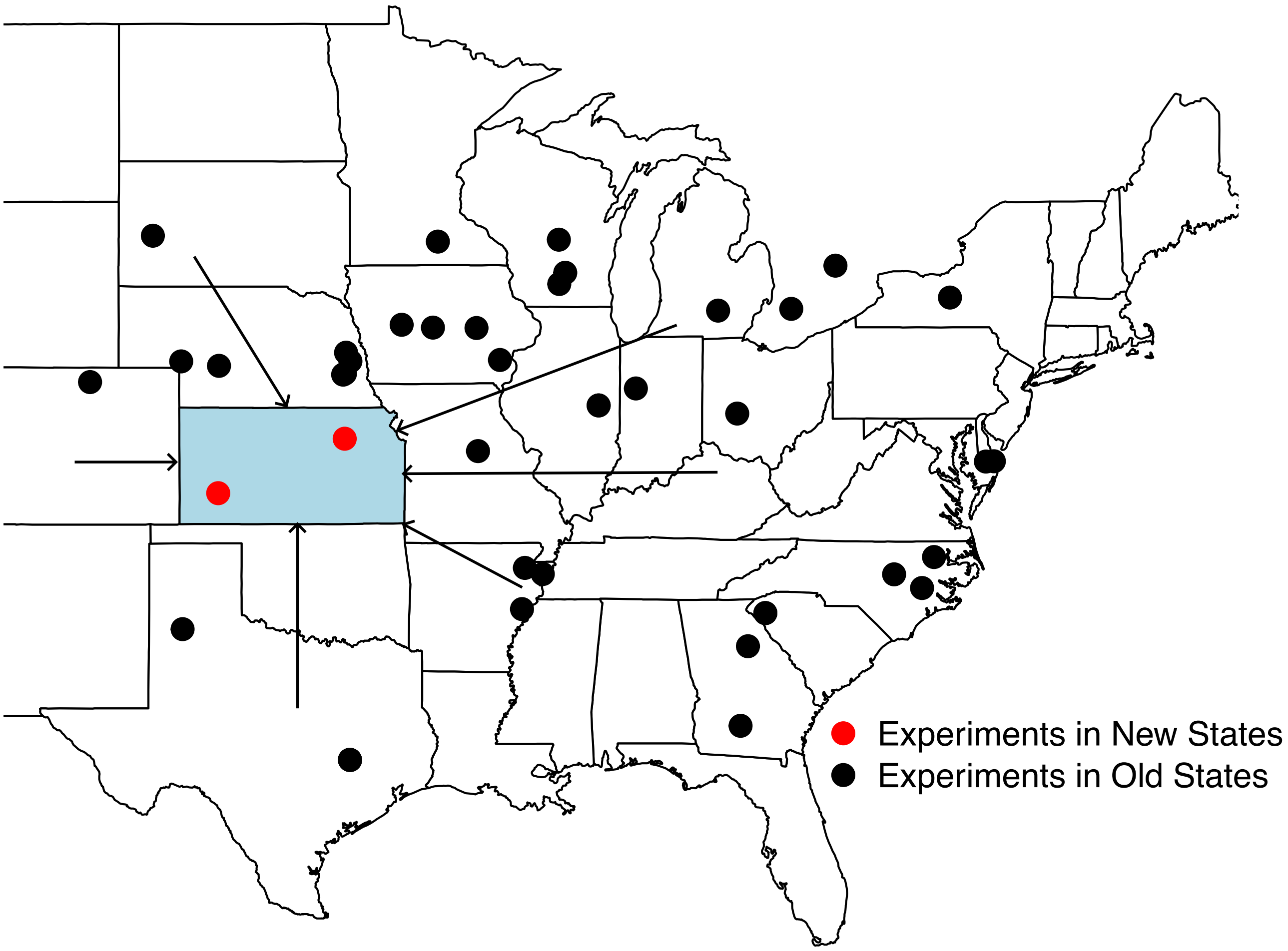

Figure S4. Graphical demonstration of the cross-validation scenario for NewState. The red circles represent experiments in new states where phenotypic data was concealed, while the black circles represent experiments in old states. The experiments in old states were used to predict the trait performance of experimental genotypes in the new states.

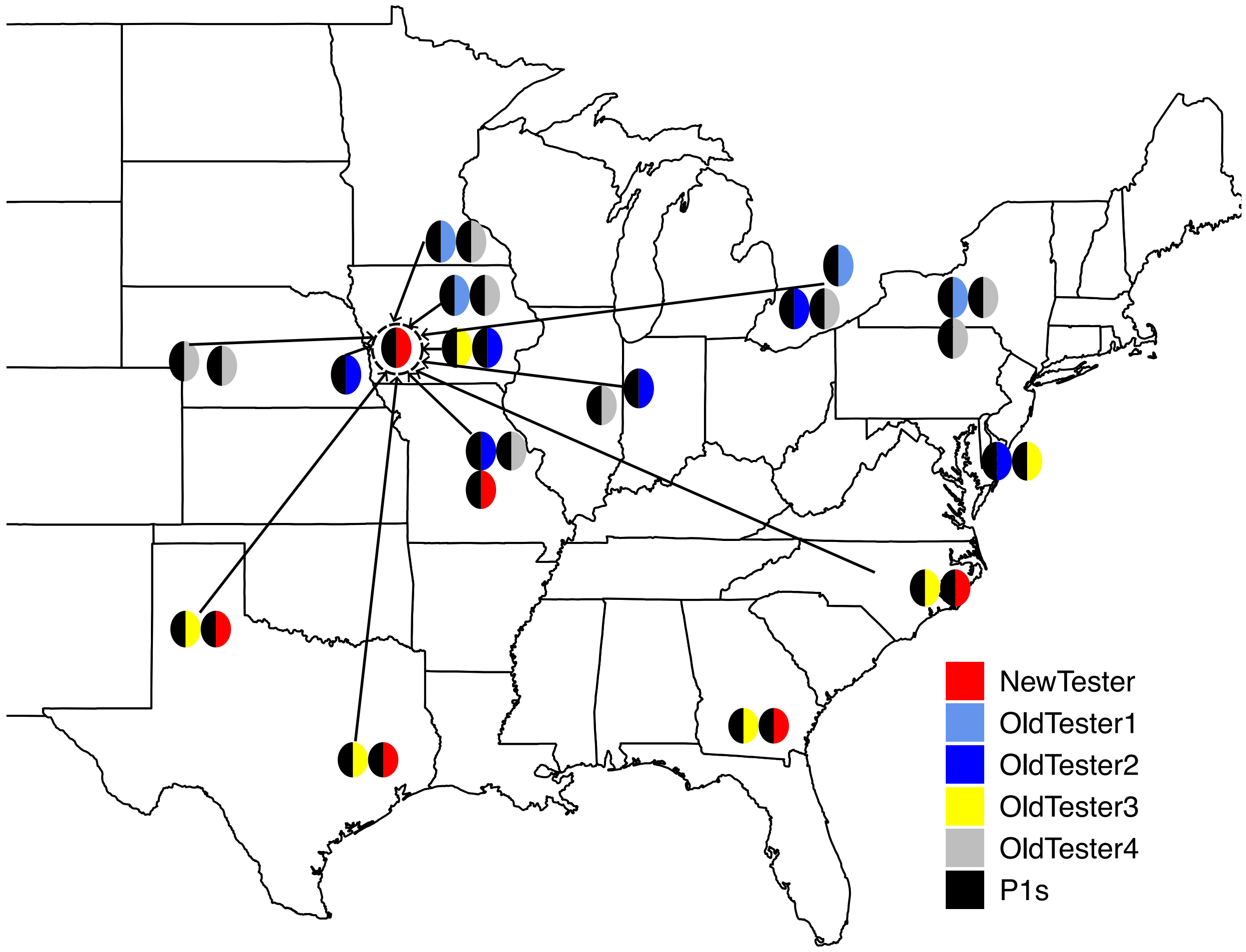

Figure S5. Graphical demonstration of the cross-validation scenario for NewTester. Each circle represents maize hybrids from a specific experiment, with the black semi-circle representing P1s and the semi-circle filled with another color representing a tester. All other old tester families are used to predict the trait performance of experimental genotypes in the new tester family, represented by the red semi-circle.

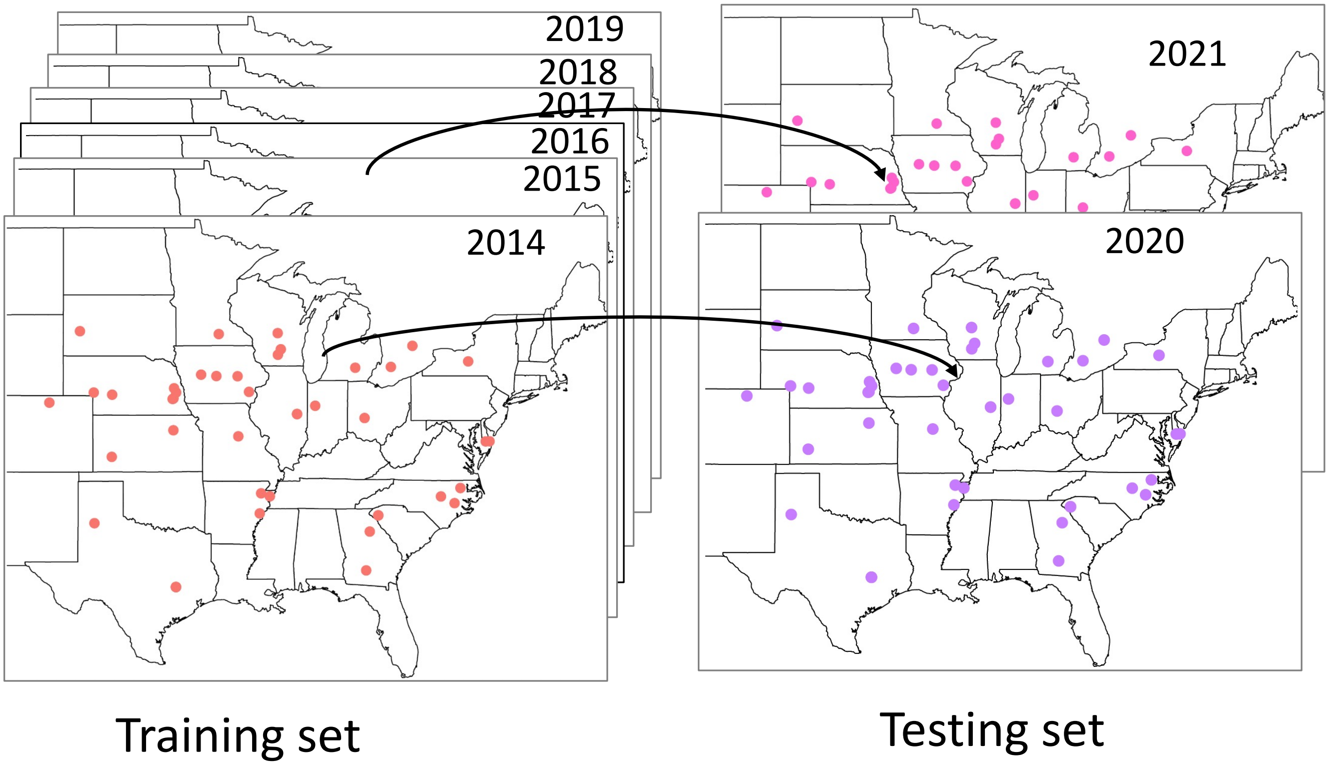

Figure S6. Graphical demonstration of the cross-validation scenario for NewGenoNewYear. All experiments were divided into four folds based on two-year intervals: 2014-2015, 2016-2017, 2018-2019, and 2020-2021. This example shows the use of all experiments from 2014 to 2019 to predict new genotypes evaluated in 2020 and 2021.

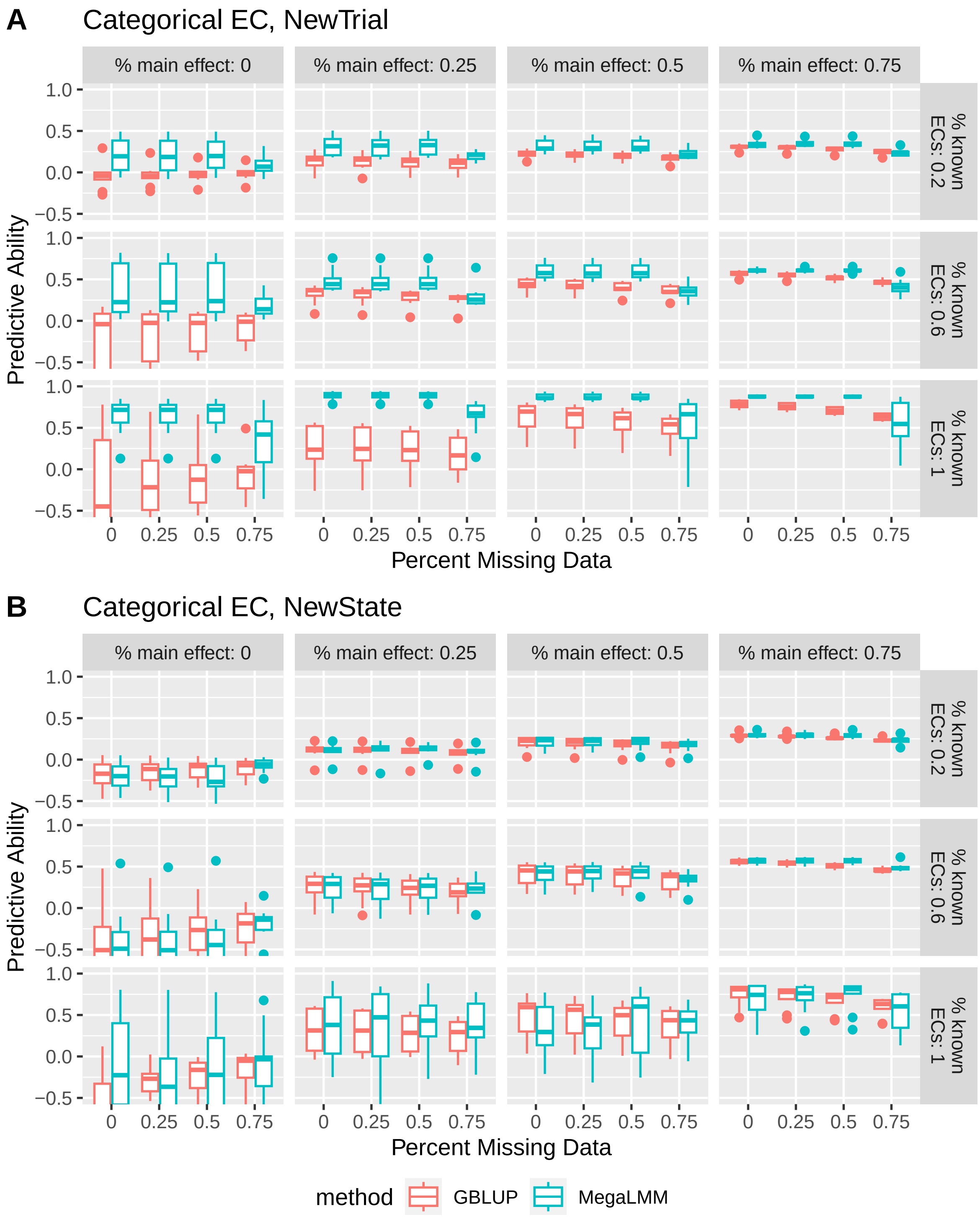

Figure S7. Predictive ability of MegaLMM and GBLUP in new environments in simulations with categorical ECs. Boxplots show predictive abilities across 10 simulations of each parameter setting. *% main effect* refers to the relative contribution of the positive covariance term across all environments, and a covariance term specifying negative covariances among groups of trials. Each scenario included 500 genotypes and 400 trials. A) Average predictive abilities of old genotypes across the 50 trials that shared a level of the grouping variable (*i.e.* "State") with the training trials. B) Average predictive abilities of old genotypes across the 50 trials that had a new level of the grouping variable (*NewState*), and therefore the value of the environmental factor in these trials could not be effectively predicted by the categorical EC. Results are shown for MegaLMM (blue) and GBLUP (red).

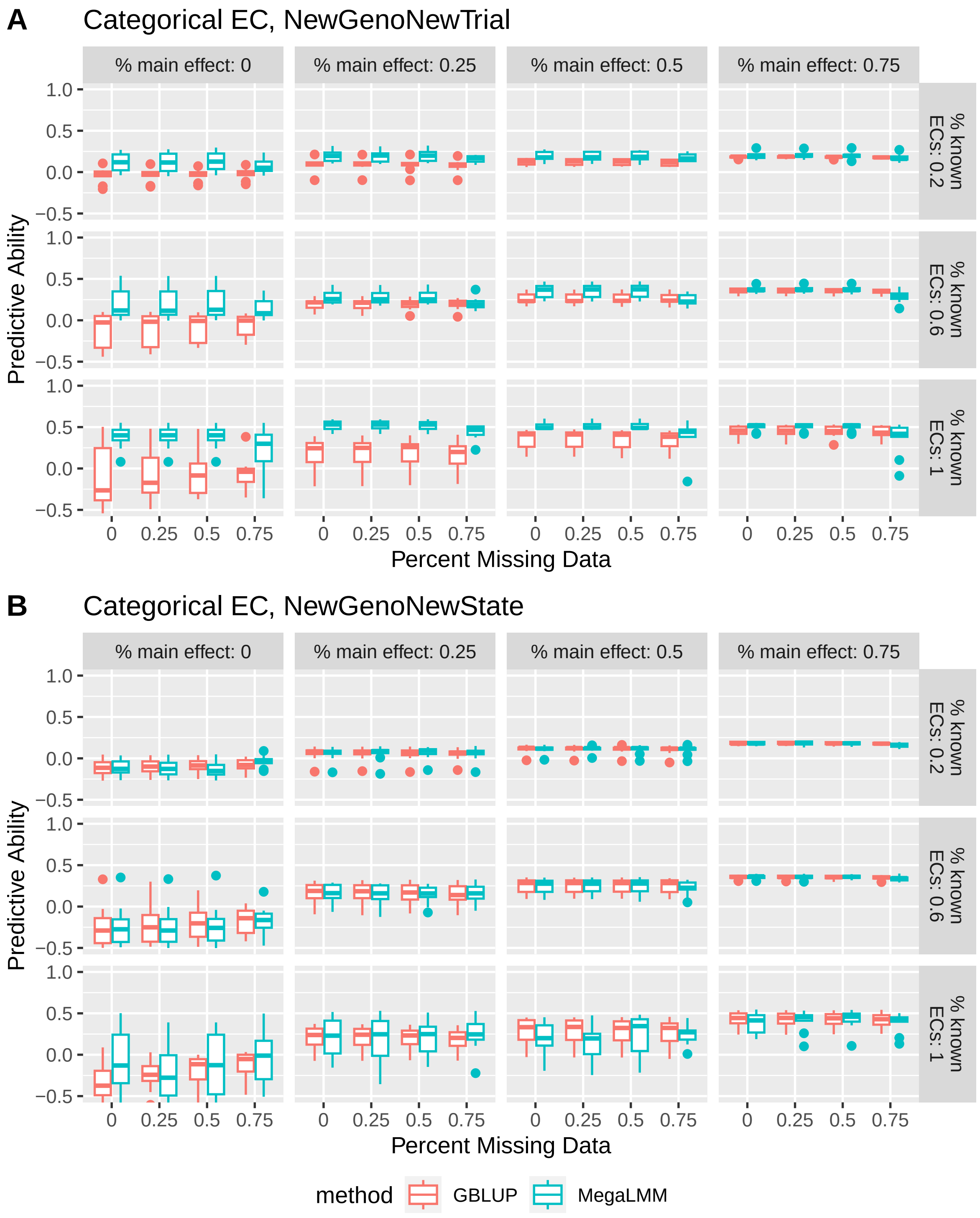

Figure S8. Predictive ability of MegaLMM and GBLUP for new genotypes in new environments in simulations with categorical ECs. Boxplots show predictive abilities across 10 simulations of each parameter setting. *% main effect* refers to the relative contribution of the positive covariance term across all environments, and a covariance term specifying negative covariances among groups of trials. Each scenario included 500 genotypes and 400 trials. A) Average predictive abilities of new genotypes across the 50 trials that shared a level of the grouping variable (*i.e.* “State") with the training trials. B) Average predictive abilities of new genotypes across the 50 trials that had a new level of the grouping variable (*NewState*), and therefore the value of the environmental factor in these trials could not be effectively predicted by the categorical EC. Results are shown for MegaLMM (blue) and GBLUP (red).

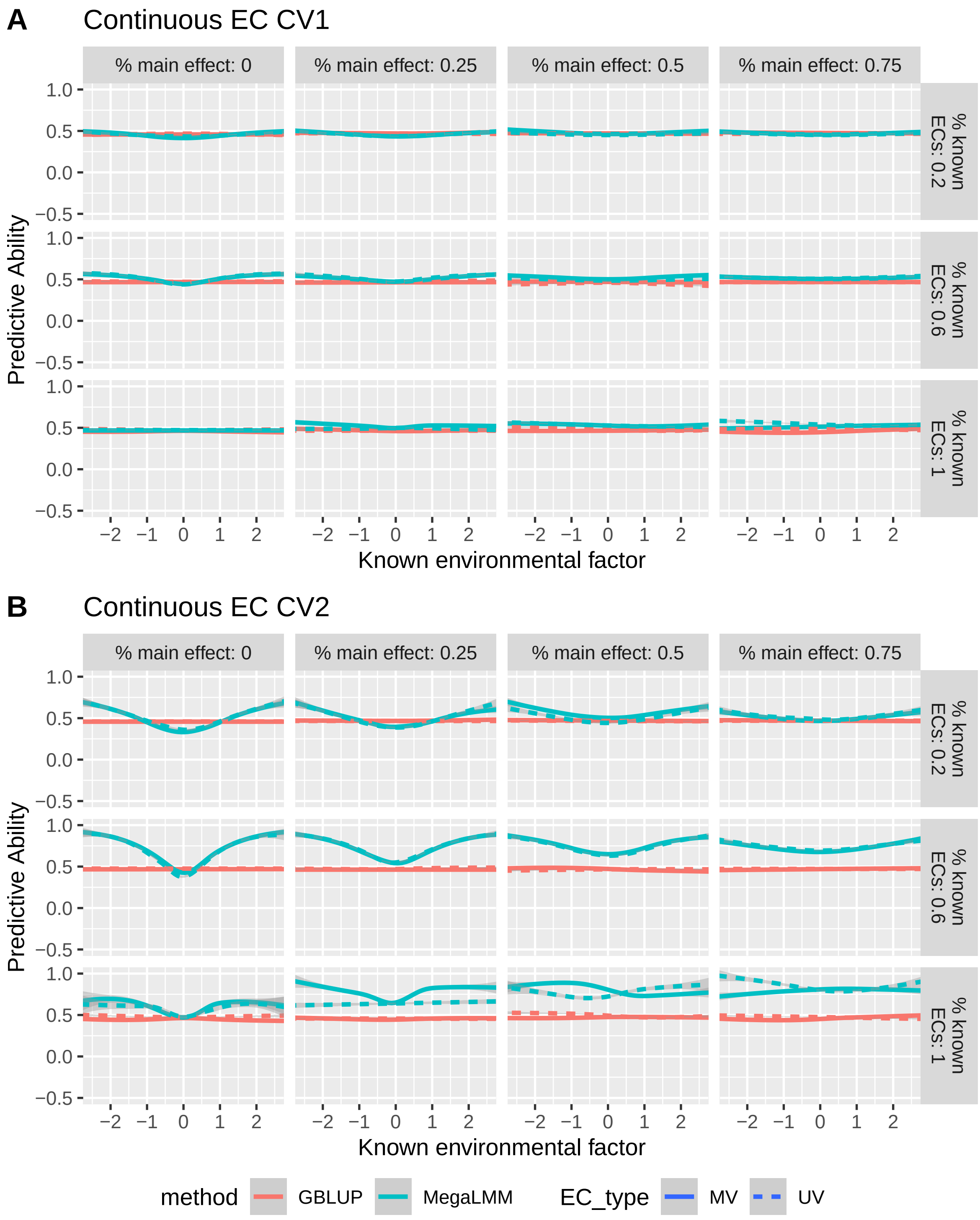

Figure S9. Predictive ability of MegaLMM and GBLUP in training environments in simulations with quantitative ECs. Boxplots show predictive abilities across 10 simulations of each parameter setting. *% main effect* refers to the relative contribution of the positive covariance term across all environments, and a covariance term specifying negative covariances among environments due to a single "known" environmental factor. Each scenario included 500 genotypes and 400 trials. Results are shown for MegaLMM (blue) and GBLUP (red). Lines were calculated using *geom_smooth* along the value of the "known" environmental variable. This variable was either directly provided to MegaLMM as a single EC (*UV*, dashed lines), or was correlated with 10 ECs provided to MegaLMM (*MV*, solid lines). A) Average predictive abilities of new genotypes across the 400 trials. B) Average predictive abilities of old genotypes across the 400 trials. Results are shown for a sparse testing scenario where 75% of genotype:trial combinations were set to missing. Predictive abilities in each trial were calculated only for genotypes that were not observed in that trial but were observed in other trials.

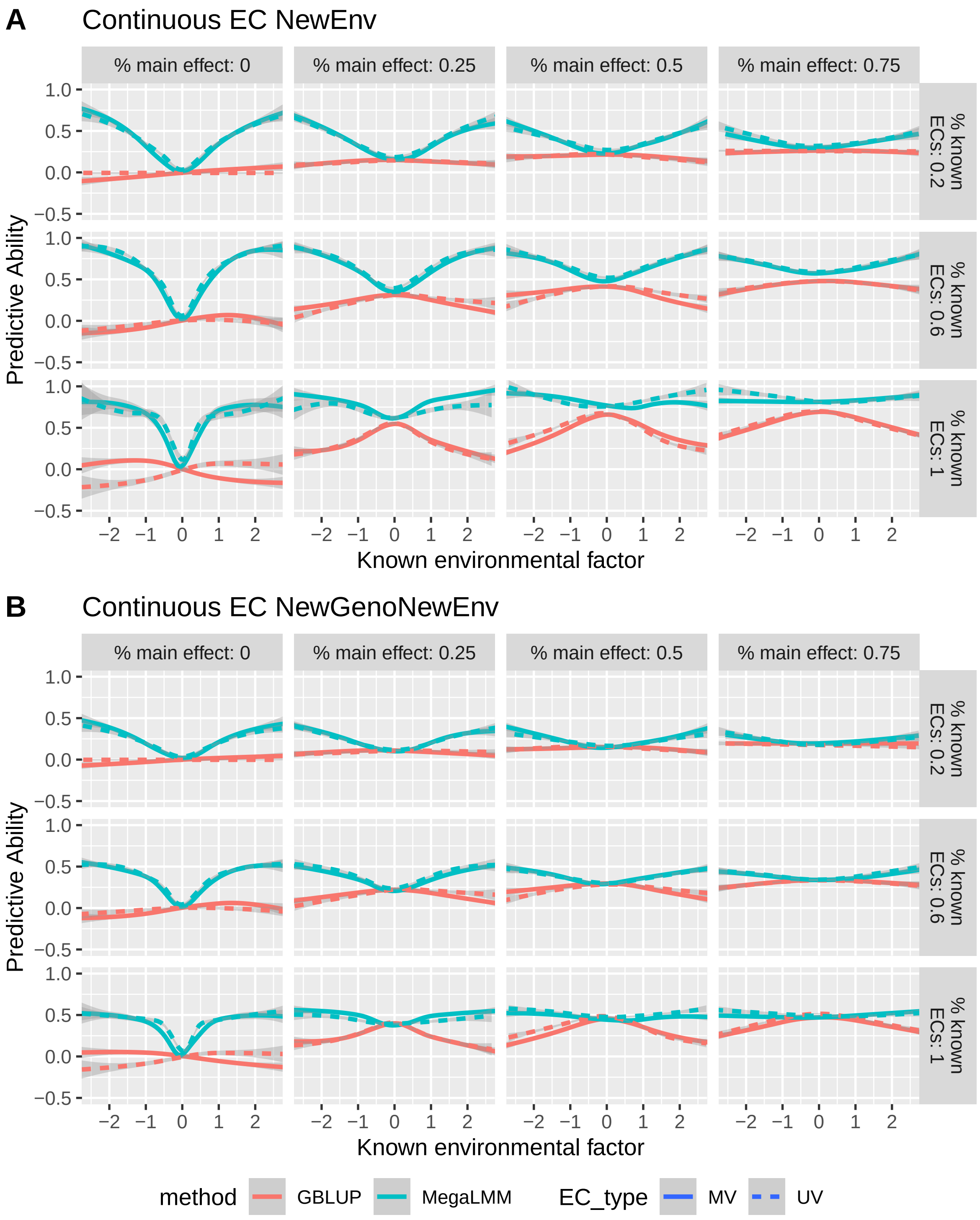

Figure S10. Predictive ability of MegaLMM and GBLUP in new environments in simulations with quantitative ECs. Boxplots show predictive abilities across 10 simulations of each parameter setting. % *main effect* refers to the relative contribution of the positive covariance term across all environments, and a covariance term specifying negative covariances among environments due to a single "known" environmental factor. Each scenario included 500 genotypes, 400 trials, and 100 new environments. Results are shown for MegaLMM (blue) and GBLUP (red). Lines were calculated using *geom_smooth* along the value of the "known" environmental variable. This variable was either directly provided to MegaLMM as a single EC (*UV*, dashed lines), or was correlated with 10 ECs provided to MegaLMM (*MV*, solid lines). A) Average predictive abilities of old genotypes across the 100 new environments. B) Average predictive abilities of new genotypes across the 100 new environments. Results are shown for a sparse testing scenario where 75% of genotype:trial combinations were set to missing. Predictive abilities in each trial were calculated only for genotypes that were not observed in that trial but were observed in other trials.

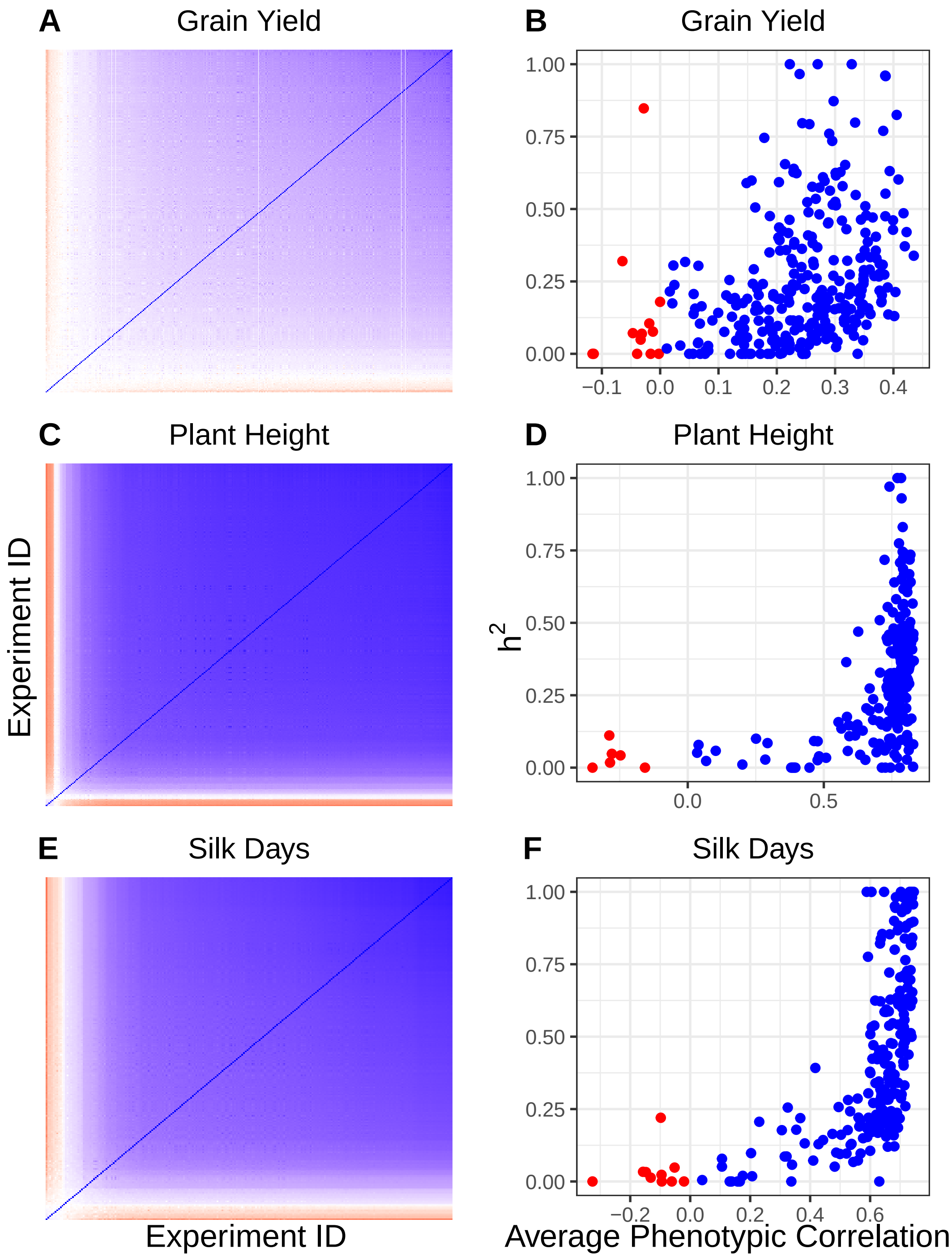

Figure S11. **Pairwise phenotypic correlation between experiments estimated by MegaLMM and the average phenotypic correlation of each experiment with all others, plotted against their narrow-sense heritability estimated by rrBLUP, for three agronomic traits (Silk Days, Plant Height, and Grain Yield).**

Table S1 Description of the 11 weather environmental variables used in our study

Parameter	Units	Long Name/Description
T2MWET	C	Wet Bulb Temperature at 2 Meters
QV2M	g/kg	Specific Humidity at 2 Meters
RH2M	%	Relative Humidity at 2 Meters
T2M_MAX	C	Temperature at 2 Meters Maximum
ALLSKY_SFC_SW_DWN	$MJ/m^2/day$	All Sky Surface Shortwave Downward Irradiance
PS	kPa	Surface Pressure
T2MDEW	C	Dew/Frost Point at 2 Meters
WS2M	m/s	Wind Speed at 2 Meters
T2M_MIN	C	Temperature at 2 Meters Minimum
T2M	C	Temperature at 2 Meters
PRECTOTCORR	mm/day	Precipitation Corrected
